# A Fatal Case of Intermittent Fever Treated Homœopathically

**Published:** 1854-12

**Authors:** 


### A FATAL CASE OF INTERMITTENT FEVER TREATED HOMŒOPATHICALLY.

The *Medical Monthly*, of New York, notices a case lately made public in a pamphlet, in which a girl, in Brooklyn, was treated for intermittent fever two months, by a homœopathic physician, and finally died from convulsions and hemorrhage of the lungs. The death occurred at a time when the parents were assured by the physician that their child was getting well "The evidence," says the writer in the *Medical Monthly*, "most clearly and unequivocally establishes that this child fell a victim to an attack of intermittent fever of no great severity, and which, humanly speaking *could have proved fatal* only by being allowed to run its course without any interference whatever." The homœopathist in charge of the patient, Dr. Parkhurst P. Wells, being asked by the Court as to the source of the hemorrhage, replied, that "it *might have come from rupture of the longitudinal sinus, coming through the ethmoid plate!*" The next day he was again in Court and testified that "the blood, if proceeding from a rupture of the longitudinal sinus, might escape through the cribriform plate of the ethmoid bone into the nasal cavities! This only in case of the softening of the brain *and its membranes!* The brain *and its membranes* being softened, I see no difficulty in a certain amount of pressure forcing the blood through the opening in that bone!" Professors Parker and Gilman were examined before the jury of inquest, and expressed doubts as to the possibility of such a hemorrhage. The homœopathist, nothing daunted by the exposure of his ignorance, professed to be greatly surprised that gentlemen holding such positions in the profession as the New York professors should be ignorant of the fact, that hemorrhage from the longitudinal sinus might take place through the ethmoid bone! The assurance of Dr. Wells truly is only equalled by his stupidity.

